# Concurrent Wernicke Encephalopathy and Posterior Reversible Encephalopathy Syndrome Following Gastric Sleeve Surgery

**DOI:** 10.31486/toj.25.0009

**Published:** 2025

**Authors:** Annika L. Chang, Doowon Huh, Kirsten Winter, Can Kocasarac

**Affiliations:** ^1^ University of Pittsburgh School of Medicine, Pittsburgh, PA; ^2^ Department of Ophthalmology, University of Pittsburgh Medical Center Vision Institute, Pittsburgh, PA; ^3^ Department of Ophthalmology, Duke University Medical Center, Durham, NC

**Keywords:** *Ophthalmoplegia*, *posterior leukoencephalopathy syndrome*, *thiamine deficiency*, *Wernicke encephalopathy*

## Abstract

**Background:**

Wernicke encephalopathy—an uncommon and severe condition caused by thiamine deficiency—is most often associated with alcohol use but can occur in other settings of nutrient deficiency. Posterior reversible encephalopathy syndrome is an acute neurologic condition characterized by reversible subcortical vasogenic edema that is often associated with hypertension. We present the case of a patient with concurrent Wernicke encephalopathy and posterior reversible encephalopathy syndrome.

**Case Report:**

A 26-year-old female with a surgical history of laparoscopic sleeve gastrectomy performed 4 months prior presented with ataxia, confusion, bilateral blurred vision, and headache. Initial examination showed reduced visual acuity (20/200 in both eyes), ophthalmoplegia, high-frequency bilateral upbeat and mild horizontal nystagmus, bilateral optic disc swelling with disc hemorrhage, and intraretinal hemorrhages. She was found to have thiamine deficiency resulting in Wernicke encephalopathy, as well as bilateral frontal, parietal, and occipital T2 hyperintensities on magnetic resonance imaging consistent with posterior reversible encephalopathy syndrome. After treatment with pulse dose thiamine repletion and antihypertensives, the patient improved clinically, with increased visual acuity (20/30 in both eyes) and complete resolution of bilateral optic disc edema and intraretinal hemorrhages. However, upbeat nystagmus remained.

**Conclusion:**

Neuro-ophthalmic signs may be early indicators of Wernicke encephalopathy and posterior reversible encephalopathy syndrome, underscoring the vital role of eye care providers in recognizing these conditions, particularly in patients who have undergone bariatric surgery. Without a high index of suspicion, Wernicke encephalopathy may be overlooked in these patients.

## INTRODUCTION

Wernicke encephalopathy is a severe, yet reversible condition caused by an acute thiamine deficiency. While Wernicke encephalopathy is known for the classic triad presentation of altered mental status, ataxia, and ocular dysfunction, only a minority of patients present with the complete triad.^1^ Autopsy-based studies have reported Wernicke encephalopathy lesions to be present in approximately 0.8% to 2.8% of the general population.^2^

Posterior reversible encephalopathy syndrome is an acute neurologic condition that presents with a wide spectrum of clinical and radiographic findings, including headache, altered mental status, visual disturbances, and seizures.^3^ Epidemiologic data show that posterior reversible encephalopathy syndrome has been diagnosed in almost all age groups, with a slight prevalence in female patients.^4^

Because both conditions present with a wide range of ophthalmic and neurologic symptoms, diagnosis is usually by exclusion. Awareness of the possible neuro-ophthalmic findings associated with Wernicke encephalopathy and posterior reversible encephalopathy syndrome is crucial for prompt diagnosis and treatment to avoid permanent ophthalmic and neurologic damage.

In this report, we describe an adult patient who developed Wernicke encephalopathy and posterior reversible encephalopathy syndrome after gastric sleeve surgery.

## CASE REPORT

A 26-year-old female with a surgical history of laparoscopic sleeve gastrectomy 4 months prior initially presented to an outside hospital emergency department (ED) with concerns of tachycardia, hypertension, and dizziness. The patient reported a 1-week history of dizziness with occasional spinning sensations; a dull, intermittent headache; and bilateral blurred vision. Computed tomography (CT) of the brain without contrast was unremarkable. At the time, the patient's dizziness and tachycardia were thought to be related to postural tachycardia and dehydration secondary to her recent history of multiple urinary tract infections for which the patient had undergone ureteral stent placement 4 days prior. The patient was advised to increase oral hydration and to follow up with an ophthalmologist to evaluate her blurred vision.

The following day, the patient was referred back to the outside hospital ED by her ophthalmologist because of bilateral optic disc edema with hemorrhage and new concerns of bilateral lower extremity weakness, difficulty walking, and mild headache. Her reported blood pressure at the time was 171/118 mm Hg. Initial physical examination findings were positive for blurriness in the right eye, inability to look left and right, and vertical nystagmus. Other findings included generalized abdominal pain and 4/5 strength in the bilateral lower extremities. CT angiography of the head and neck and CT of the head were normal, with no signs of large vessel occlusion or stroke. Cerebrospinal fluid (CSF) studies from lumbar puncture were unremarkable, but no opening pressure was obtained. The patient was given intravenous (IV) labetalol and was transferred to our institution for further neurologic and ophthalmologic evaluation.

**Figure 1.**
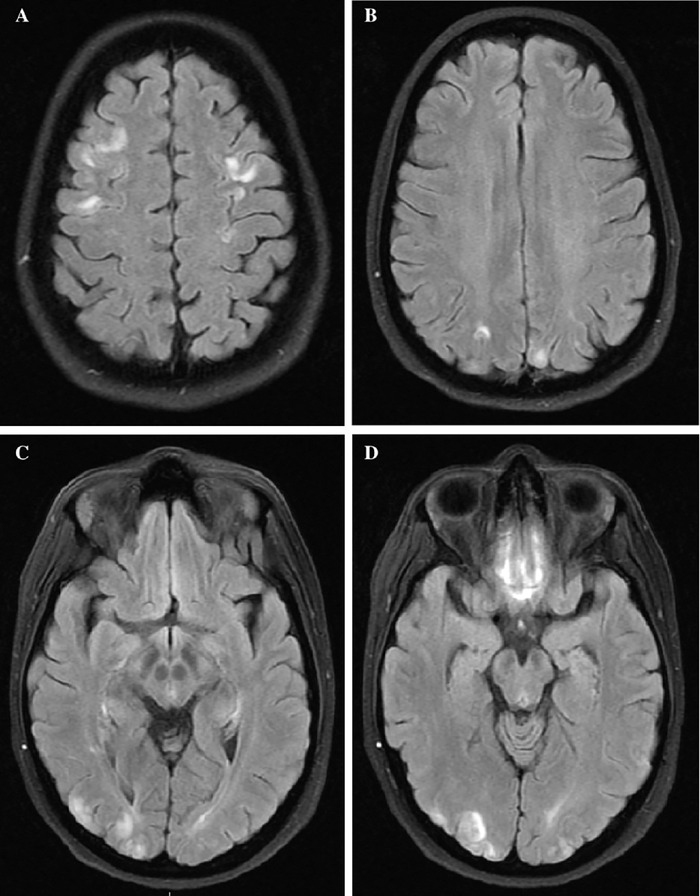
Magnetic resonance imaging axial T2-weighted fluid-attenuated inversion recovery with fat suppression images show multiple cortical and subcortical hyperintensities in the (A) bilateral parietal lobes, (B) parieto-occipital region, (C) bilateral occipital lobes, and (D) bilateral frontal lobes suggestive of posterior reversible encephalopathy syndrome.

On arrival at our institution's ED, the patient was tachycardic to 109 beats per minute, hypertensive at 124/101 mm Hg, and saturating at 98% on room air. The patient's laboratory workup was notable for an elevated erythrocyte sedimentation rate of 58 mm/h (reference range, <20 mm/h) and C-reactive protein of 12.8 mg/dL (reference range, <1 mg/dL), with a normal vitamin B12 level of 698 pg/mL (reference range, 200-900 pg/mL). Magnetic resonance imaging (MRI) of the brain and orbits with and without contrast showed bilateral frontal, parietal, and occipital T2 hyperintensities concerning for posterior reversible encephalopathy syndrome (Figure 1). Magnetic resonance angiography of the head and neck and magnetic resonance venography were unremarkable. The patient was admitted to the neurology service.

Ophthalmology was consulted because of the patient's acute painless bilateral vision loss, altered mental status, and bilateral optic disc edema. During the initial consult, the patient reported a 1-week history of progressively worsening bilateral vision loss that she described as “looking through water,” as well as a 4-day history of being unable to abduct and adduct both eyes, with limited upward and downward eye movements. The patient also noted an ongoing history of bilateral hearing loss. She denied any history of eye trauma, procedures, or surgeries.

The initial examination showed visual acuities of 20/200 in both eyes. Other noteworthy findings included diminished color vision; extraocular eye movement deficits related to abduction (–4), adduction (–4), downgaze (–2), and upgaze (–1) in both eyes; and high-frequency bilateral upbeat nystagmus with mild horizontal nystagmus. The posterior segment of both eyes showed 360-degree optic disc swelling with disc hemorrhage along the superior and inferior arcades, as well as intraretinal hemorrhages that were worse in the right eye than the left. The macula and periphery in both eyes appeared normal. Intraocular pressure and anterior segment examination in each eye were normal.

The wide range of this patient's symptoms resulted in a broad differential, including neurovascular, vitamin deficiency, infectious, and autoimmune etiologies. Given the patient's clinical features (headache, altered mental status, blurred vision, weakness), acute hypertension, and T2 hyperintensities on MRI brain imaging, she was diagnosed with posterior reversible encephalopathy syndrome.

With a goal of normotension, the patient was started on 2.5 mg amlodipine daily for hypertensive control, and the dosage was uptitrated to 5 mg daily the following day. A thiamine level was ordered because of the patient's recent gastric sleeve surgery, and she was started on an empiric 3-day pulse therapy 500 mg dose of IV thiamine supplementation.

The infectious etiology (eg, toxoplasmosis, Lyme disease, *Bartonella*, herpes simplex virus, tuberculosis) was included in the initial differential because of the patient's recent history of multiple infections. After a repeat lumbar puncture, CSF studies were sent for cytology, neuromyelitis optica, infectious, and autoimmune encephalopathy panels. Serum studies were sent for neuromyelitis optica, myelin oligodendrocyte glycoprotein, antineutrophil cytoplasmic antibody, and serum autoimmune encephalopathy panels. Chest x-ray showed no abnormal findings. Per the neurology team, the patient was started on a 5-day pulse therapy course of 1,000 mg IV methylprednisolone.

On the second day of admission, the patient's thiamine level results revealed a low level of 21 nmol/L (reference range, 70-180 nmol/L), and she remained on 500 mg IV thiamine. After 3 days of 500 mg IV thiamine, the patient's thiamine supplementation was tapered to 250 mg IV for 3 days, followed by 100 mg daily oral thiamine.

On the third day of admission, the patient's CSF laboratory workup was negative for Venereal Disease Research Laboratory test, herpes simplex virus, Epstein-Barr virus, varicella-zoster virus, and cryptococcus. Moreover, the CSF studies from the patient's lumbar puncture showed no signs of meningitis, making an infectious cause less likely on the differential.

On the fourth day of admission, the patient was seen by a hospital dietician who learned that the patient's home diet included high protein with low sugar. After her gastric sleeve surgery, the patient initially took vitamin B12, vitamin D, and calcium. However, she said she had difficulty tolerating these supplements and had discontinued them. The patient's weight prior to gastric sleeve surgery was 112 kg, and her weight on admission was 78 kg, a 34 kg weight loss in 4 months.

Following her 5-day course of IV methylprednisolone, the patient was started on a daily 60 mg dose of oral prednisone.

The patient was diagnosed with Wernicke encephalopathy because of her history of gastric sleeve surgery, rapid weight loss, examination findings (ie, altered mental status, ataxia, ocular dysfunction), and low thiamine level. This diagnosis was confirmed by her clinical improvement after thiamine supplementation.

Throughout her hospitalization, the patient's ocular dysfunctions gradually improved. By day 5 of hospitalization, her visual acuity had improved to 20/100 in both eyes, and she had complete recovery of color vision, improved abduction and adduction of both eyes, and resolving bilateral optic disc edema. Horizontal nystagmus and upbeat nystagmus were unchanged.

Repeat MRI of the brain performed 6 days after her initial imaging showed stable T2 hyperintensities with improvement in contrast enhancement. Other potential autoimmune etiologies were less likely, given that the patient's neuromyelitis optica, myelin oligodendrocyte glycoprotein, and serum/CSF autoimmune encephalopathy panels were negative. However, Lyme disease immunoglobulin G with 5 Western blot bands was positive. Although Lyme disease immunoglobulin M was negative, the patient was started on 100 mg oral doxycycline twice daily for 21 days.

**Figure 2.**
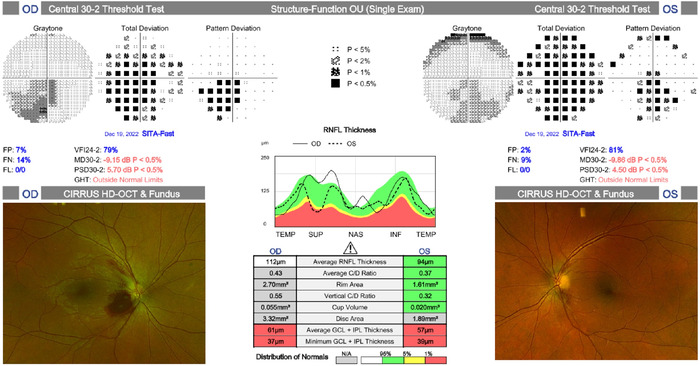
Humphrey visual field 30-2 and optical coherence tomography tests with fundus photos completed on day 8 of admission show optic disc edema with hemorrhages in the right eye and central scotoma in both eyes.

A Humphrey visual field 30-2 test performed 8 days after admission demonstrated abnormal visual fields with inferior scotoma not respecting the vertical meridian (Figure 2). Optical coherence tomography showed supranormal retinal nerve fiber layer thickness of the right eye, normal retinal nerve fiber layer thickness of the left eye, and normal macular findings in both eyes (Figure 2).

**Figure 3.**
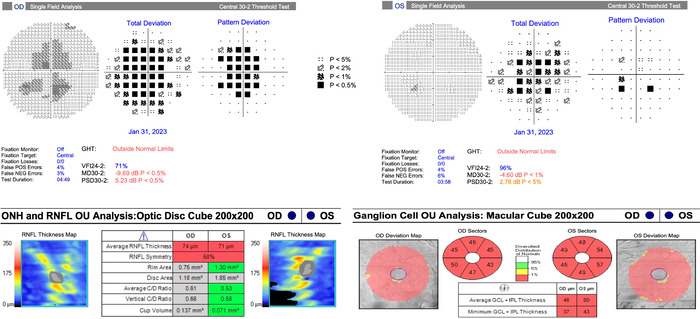
Humphrey visual field 30-2 and optical coherence tomography tests completed at 1-month follow-up show reduced retinal nerve fiber layer and ganglion cell layer thickness.

The patient was discharged 9 days after admission to inpatient rehabilitation with outpatient neurology, neuroimmunology, and ophthalmology follow-up. At the patient's 1-month ophthalmology follow-up, her visual acuity had improved to 20/60 in the right eye and 20/70 in the left eye. Fundus examination showed trace temporal pallor of both optic discs but complete resolution of bilateral optic disc edema and intraretinal hemorrhages. Optical coherence tomography showed reduced retinal nerve fiber layer and ganglion cell layer thickness (Figure 3). The patient's upbeat nystagmus was unchanged. By the 4-month follow-up, visual acuity had improved to 20/30 in each eye, with an improved but persistent central/inferior scotoma in the right eye and resolution of the visual field defect in the left eye.

## DISCUSSION

Both Wernicke encephalopathy and posterior reversible encephalopathy syndrome present as severe, acute neurologic conditions with possible ocular involvement that require early detection and treatment to prevent long-term neurologic and ophthalmic sequelae.

While our patient's symptoms were broad, she had findings concerning for Wernicke encephalopathy (altered mental status, ataxia, ocular dysfunction) and a history of gastric sleeve surgery that resulted in a 30% weight reduction in 4 months. Moreover, the patient experienced difficulties tolerating vitamin B12, vitamin D, and calcium supplements during this time, which might have affected her overall nutritional intake. After a bariatric procedure, the average timeline for Wernicke encephalopathy symptom presentation in patients with thiamine deficiency is usually 4 to 12 weeks because of limited tissue storage and the water-soluble nature of thiamine.^5^ Our patient's low thiamine level, a possible complication of sleeve gastrectomy, and her rapid weight loss may have contributed to her development of Wernicke encephalopathy.^6,7^ Moreover, while the patient's bilateral optic disc edema and retinal hemorrhages are rarer ophthalmic symptoms of Wernicke encephalopathy, these fundoscopic findings were reported in a 37-year-old female who underwent sleeve gastrectomy.^8^ Prevalence rates for optic disc edema and retinal hemorrhages have been reported to be as low as 4% and 2%, respectively, in patients with Wernicke encephalopathy.^9^ However, studies suggest that these findings are likely underestimated as not all patients with Wernicke encephalopathy undergo fundoscopic examination by an ophthalmologist.^1,8^ Current recommendations for Wernicke encephalopathy treatment include empiric high-dose IV thiamine, followed by oral thiamine supplementation.^10^

Our patient also had symptoms concerning for posterior reversible encephalopathy syndrome, given her altered mental status, hypertension, headaches, and visual disturbances, as well as bilateral frontal, parietal, and occipital T2 hyperintensities. While the exact pathophysiologic mechanism of posterior reversible encephalopathy syndrome remains unknown, early literature suggested hypotheses involving cerebral hyperperfusion syndrome and endothelial dysfunction as the cause of the reversible vasogenic subcortical edema localized to the parietal and occipital lobes.^11,12^ To prevent irreversible neurologic and ophthalmic damage, early diagnosis of posterior reversible encephalopathy syndrome with MRI is crucial. T2 hyperintensities in a distinctive parieto-occipital pattern, as seen on our patient's MRI, are the principal neuroimaging findings for posterior reversible encephalopathy syndrome.^13^ Treatment for posterior reversible encephalopathy syndrome focuses on treating the underlying cause, which in the case of our patient included strict blood pressure control to prevent further hypertensive episodes.^4^

Although our patient had positive risk factors and neurologic examination findings consistent with Wernicke encephalopathy, the diagnosis was delayed. The diagnostic delay can be attributed to several factors, including the patient's recent history of multiple infections, the initial presentation of multiple symptoms, the coexisting presentation of posterior reversible encephalopathy syndrome, and the rarity of Wernicke encephalopathy in a patient with no history of heavy alcohol use. Moreover, although the patient had a history of bariatric surgery, symptom onset 4 months following her surgery varies from the average timeline of Wernicke encephalopathy symptom presentation. This case demonstrates the importance of maintaining a high index of suspicion for Wernicke encephalopathy for high-risk groups, including patients with recent bariatric surgery or a history of rapid weight loss.

Although Wernicke encephalopathy and posterior reversible encephalopathy syndrome have been studied separately in the literature, we found only 1 case reporting concurrent Wernicke encephalopathy and posterior reversible encephalopathy syndrome, and the patient had acute graft-versus-host disease.^13^ Given that both conditions feature overlapping symptom presentations, including both conditions in the differential diagnosis is critical.

## CONCLUSION

For this patient with findings of Wernicke encephalopathy and posterior reversible encephalopathy syndrome, treatment of both underlying conditions was critical for clinical improvement. Clinicians must keep a broad differential with the understanding that multiple nonspecific findings could indicate 2 simultaneous disease processes. Further studies would help determine if the pathophysiology of these conditions is related.

## Notes

*This article meets the Accreditation Council for Graduate Medical Education and the American Board of Medical Specialties Maintenance of Certification competencies for Patient Care and Medical Knowledge.*

## References

[1] Harper CG, Giles M, Finlay-Jones R. Clinical signs in the Wernicke-Korsakoff complex: a retrospective analysis of 131 cases diagnosed at necropsy. J Neurol Neurosurg Psychiatry. 1986;49(4):341-345. doi: 10.1136/jnnp.49.4.341 3701343 PMC1028756

[2] Harper C, Fornes P, Duyckaerts C, Lecomte D, Hauw JJ. An international perspective on the prevalence of the Wernicke-Korsakoff syndrome. Metab Brain Dis. 1995;10(1):17-24. doi: 10.1007/BF01991779 7596325

[3] Ando Y, Ono Y, Sano A, Fujita N, Ono S. Posterior reversible encephalopathy syndrome: a review of the literature. Intern Med. 2022;61(2):135-141. doi: 10.2169/internalmedicine.7520-21 34275982 PMC8851194

[4] Fischer M, Schmutzhard E. Posterior reversible encephalopathy syndrome. J Neurol. 2017;264(8):1608-1616. doi: 10.1007/s00415-016-8377-8 28054130 PMC5533845

[5] Singh S, Kumar A. Wernicke encephalopathy after obesity surgery: a systematic review. Neurology. 2007;68(11):807-811. doi: 10.1212/01.wnl.0000256812.29648.86 17353468

[6] Whitfield KC, Bourassa MW, Adamolekun B, Thiamine deficiency disorders: diagnosis, prevalence, and a roadmap for global control programs. Ann N Y Acad Sci. 2018;1430(1):3-43. doi: 10.1111/nyas.13919 30151974 PMC6392124

[7] Auge M, Menahem B, Savey V, Lee Bion A, Alves A. Long-term complications after gastric bypass and sleeve gastrectomy: what information to give to patients and practitioners, and why? J Visc Surg. 2022;159(4):298-308. doi: 10.1016/j.jviscsurg.2022.02.004 35304081

[8] Serlin T, Moisseiev E. Fundus findings in Wernicke encephalopathy. Case Rep Ophthalmol. 2017;8(2):406-409. doi: 10.1159/000478924 28924437 PMC5597920

[9] Isen DR, Kline LB. Neuro-ophthalmic manifestations of Wernicke encephalopathy. Eye Brain. 2020;12:49-60. doi: 10.2147/EB.S234078 32636690 PMC7335288

[10] Chandrakumar A, Bhardwaj A, 't Jong GW. Review of thiamine deficiency disorders: Wernicke encephalopathy and Korsakoff psychosis. J Basic Clin Physiol Pharmacol. 2018;30(2):153-162. doi: 10.1515/jbcpp-2018-0075 30281514

[11] Hinchey J, Chaves C, Appignani B, A reversible posterior leukoencephalopathy syndrome. N Engl J Med. 1996;334(8):494-500. doi: 10.1056/NEJM199602223340803 8559202

[12] Bartynski WS. Posterior reversible encephalopathy syndrome, part 1: fundamental imaging and clinical features. AJNR Am J Neuroradiol. 2008;29(6):1036-1042. doi: 10.3174/ajnr.A0928 18356474 PMC8118828

[13] Di Giuliano F, Picchi E, Scaggiante J, Posterior reversible encephalopathy syndrome and Wernicke encephalopathy in patient with acute graft-versus-host disease. Radiol Case Rep. 2019;14(8):971-976. doi: 10.1016/j.radcr.2019.05.024 31193981 PMC6545363

